# Surgical Treatment of Solid Pseudopapillary Tumor in Pediatric Patients: Two Case Reports and a Brief Narrative Review

**DOI:** 10.1155/crom/8183273

**Published:** 2025-06-01

**Authors:** Dario Talloa, Simona Cordaro, Giorgio Attinà, Stefano Mastrangelo, Alberto Romano, Anna Fregola, Palma Maurizi, Lorenzo Nanni, Antonio Ruggiero

**Affiliations:** ^1^Pediatric Oncology Unit, Fondazione Policlinico Universitario A. Gemelli IRCCS, Rome, Italy; ^2^Department of Woman and Child Health and Public Health, Università Cattolica Sacro Cuore, Rome, Italy; ^3^Paediatric Surgery Unit, Department of Woman and Child Health and Public Health, Fondazione Policlinico Universitario A. Gemelli IRCCS, Rome, Italy

**Keywords:** abdominal mass, children, pancreatic surgery, SPNs

## Abstract

Solid pseudopapillary neoplasms (SPNs) represent a tumor of the exocrine pancreas, belonging to the group of pancreatic cystic neoplasms. It is exceedingly uncommon for SPNs to manifest in extrapancreatic locations, such as the mesocolon, greater omentum, jejunum, and ovary. SPNs are considered very rare, constituting about 0.17%–2.5% of all pancreatic neoplasms and 6% of cystic pancreatic neoplasms. We present two pediatric cases of SPNs: one involving a 16-year-old female patient and the other a 14-year-old girl, both diagnosed and treated in our center with surgical resection. The experience of our center confirms that wide margin surgery, with associated metastasectomy if necessary, represents the therapy of choice for SPNs, ensuring effective control of the disease.

## 1. Introduction

Solid pseudopapillary neoplasms (SPNs) of the pancreas were first described between the 1950s and 1960s but were officially recognized as a separate entity, distinct from other pancreatic tumors in 1996 by the World Health Organization (WHO). These neoplasms typically arise in young women, often less than 35 years of age (with peak incidence between the ages of 20 and 30), while they are less common in men [1–3]. They were considered exceedingly rare tumors, constituting about 0.17%–2.5% of all exocrine pancreatic neoplasms [4, 5] and 6% of cystic pancreatic neoplasms [6] but a rise in incidence has been noted in the last decades, which could be attributed to a greater awareness of this condition in the medical community and to greater accessibility to radiological examinations such as abdomen CT scans [1].

They are considered low-grade malignant tumors of the pancreas characterized histologically by poorly cohesive polygonal epithelial elements organized into pseudopapillary structures. The tumor is characterized by multiple capillary vessels which, when cystic, are surrounded by one or more layers of surviving tumor cells, giving the lining an irregular “pseudopapillary” appearance. Tumor cells have a moderate amount of eosinophilic cytoplasm and round nucleus with finely speckled chromatin and longitudinal grooves. The presence of hyaline globules, although not specific, is a characteristic feature of these neoplasms. These globules are diastase-resistant and PASD-positive eosinophilic cytoplasmic inclusions corresponding to alpha-1-antitrypsin granules [7]. Nuclear atypia and increased mitotic figures are typically absent. In some cases, the tumor may be circumscribed by a fibrous wall or capsule that contains calcifications. Despite its considerable dimensions, vascular and perineural invasion are uncommon in SPN [8]. In contrast to larger tumors, which are composed of a combination of solid, hemorrhagic and necrotic components, smaller SPNs may be histologically homogeneous. From an immunohistochemical perspective, tumor cells typically exhibit nuclear and cytoplasmic immunoreactivity for beta-catenin, CD10, progesterone receptor, Cyclin D1, and vimentin [9–12].

The pathogenesis of SPNs is still unclear, but they are thought to derive from pluripotent stem cells. The prevalence of this condition in young women suggests the possibility of a hormonal influence on its development.

From a genetic standpoint, SPNs have key genetic mutations: almost all SPNs harbor mutations in the CTNNB1 gene, which encodes the beta-catenin protein. This determines a nuclear accumulation of beta-catenin; this mutation is helpful in distinguishing these lesions from other pancreas neoplasms, which do not usually exhibit this aberration. Many SPNs show overexpression of Cyclin D1, a downstream effector of beta-catenin [10]. Whole exome sequencing has identified many genetic variations in SPNs, including single nucleotide polymorphisms (SNPs) in genes like PKD1, USP9X, EP400, and MED12 [13]. A modern series of cases focused on searching for potentially actionable mutations and was able to find mutations in genes like PIK3CA, PTEN, CDKN2, MSH2, BRCA2, and ATRX [14].

Clinical manifestations can be heterogeneous. In more than 1/3 of cases, patients are completely asymptomatic [1]; more often, the slow growth of the neoplasm in the abdominal cavity determines nonspecific abdominal pain or cramps. The most common presenting symptoms are discomfort, abdominal pain, or abdominal swelling, present in more than 60% of patients. Patients can also present nausea or vomiting, weight loss, anorexia, fever, fatigue, or a palpable abdominal mass. Occurrence of jaundice or pancreatitis is less common (10% and 5%, respectively) [1].

They typically manifest as a solitary localized lesion with cystic, solid, or mixed aspects with calcifications in the context. More commonly, SPNs are localized in the body and tail of the pancreas; less frequently, they are found in the head [15]. Adolescents, in contrast to older patients, tend to experience SPNs mostly in the head of the pancreas [16]; nevertheless, the location of the lesion is not helpful in identifying SPNs, which can occur in any part of the pancreas. It is exceedingly uncommon for SPNs to manifest in extrapancreatic locations, such as the mesocolon, greater omentum, jejunum, and ovary. These rare forms are believed to originate from ectopic pancreatic tissue present in these sites [17–21].

It was and usually still is perceived as a low-grade malignant tumor with good prognosis: Most patients have localized disease at diagnosis, with no evidence of metastasis or local invasion. Treatment usually consists of surgical resection, and when R0 surgical resection is feasible, patients can attain prolonged periods of disease free survival [22, 23]. There is no standardized definition for malignancy in SPNs, but most researchers use criteria similar to other pancreatic neoplasms like presence of metastasis, recurrence after complete resection, and infiltration of nearby tissues; WHO suggested that histological presence of perineural invasion, angioinvasion, or deep invasion of adjacent structures could be factors in defining a malignant transition and advised the medical community to be wary of incompletely resected tumors which could hide regions were anatomopathological criteria for malignancy could have been missed [24]. SPNs have a strong avidity for ^18^F-FDG, but the mechanism of increased glucose intake is still not clearly defined; both GLUT-1 overexpression and an increase in glucose uptake mediated by SGLT1 and SGK1 because of impaired beta catenin degradation have been suggested as possible mechanisms of increase glucose uptake [25–27]. Although factors affecting prognosis are still being studied, some authors have pointed out some clinical and nonclinical elements that may correlate with survival.. In a literature review, Yepuri et al. identify the presence of lymph node positive disease and anatomopathological lymphovascular invasion (LVI) as the main predictors of recurrence [28]. In another review from 2016, Yang et al. observed a significant association between poor prognosis and a *Ki* − 67 > 4% [29].

## 2. Materials and Methods

A review of the current literature was performed in PubMed and Web of Science, with final searches completed by August 31, 2024. The main search strategy used a combination of keywords for solid pseudopapillary tumors of the pancreas in children and adolescents: (solid pseudopapillary tumor of the pancreas) AND (children or adolescent)) AND (pancreatic tumors).

Searches were conducted by manuscript authors, and articles were checked for duplicates by two separate authors. Articles were independently reviewed by two authors who selected them for inclusion based on the criteria described below.

The inclusion criteria were as follows: case reports or case series with detailed clinical data and extractable complete case information.

The exclusion criteria were as follows: repeated reports, animal experiments, secondary analysis of literature, articles with incomplete medical history, or anatomopathological findings of reported cases.

Our search showed a few secondary analyses of literature related to SPNs of the pancreas regarding the management of such cases, while most articles were case reports and case series. By analyzing the clinical data of our cases and collecting the relevant literature, this study explores and summarizes the clinical features and treatment of SPT in children. The goal is to help with the early and accurate diagnosis and treatment of SPT.

## 3. Case Report

### 3.1. Case 1

**Figure 1:**
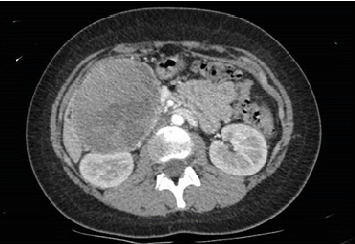
Voluminous solid expansive formation with oval morphology in the right median–paramedian subhepatic region with epicenter in the Duodenal C and maximum axial diameters of 88 × 87 mm (dAPxdTT) and craniocaudal extension of 83 mm. It compresses and displaces the pancreatic head cranially and the duodenum laterally.

**Figure 2:**
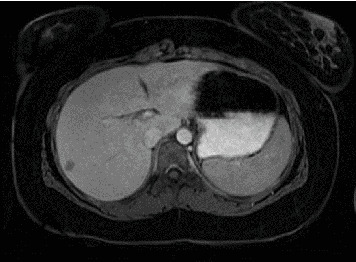
At the passage between the VII and VI segments, a localized lesion of 13 × 9 mm is documented representing secondarism.

A 16-year-old female presented to the emergency department with severe anemia (Hb 5.7 g/dL, mean corpuscular volume (MCV) 40.9 fL, hematocrit 21%,) identified while she was undergoing evaluations for a year-long history of reduced food intake with associated weight loss (−20 kg in 1 year) and chronic constipation. She was under treatment for a major depressive disorder; treatment consisted of paroxetine and olanzapine. In the emergency department, blood samples confirmed severe anemia with hypoferritinemia, and she was admitted to the pediatric ward to undergo further evaluations. Physical examination and subsequent imaging revealed a large encapsulated abdominal mass of 8.8 × 8.4 cm in the pancreatic head-body region without clear invasion of adjacent structures (Figure 1). She was then transferred to the pediatric oncology ward; a full body CT scan excluded the presence of distant metastasis; tumoral markers (AFP, beta-HCG, and CEA) and serological screening for infectious diseases were negative. She underwent an ultrasound-guided biopsy of the lesion, which led to the diagnosis of a SPN of the pancreas. Nine days later, she underwent laparotomy (inverted-Y laparotomy) with enucleation of the pancreatic tumor. Intraoperative findings showed an encapsulated mass adherent but not macroscopically infiltrating the duodenum and mesocolon. The tumor was completely resected with clear surgical margins; the mass had a maximum diameter of 9.5 cm, and three regional lymph nodes were removed for histological analysis. The surgery was conducted by pediatric surgeons of our institution after multidisciplinary discussion with adult pancreatic surgeons. Anatomopathological examination of the lesion confirmed the diagnosis of an SPN of the pancreas with diffuse positivity and aberrant nuclear expression of beta-catenin. Chromogranin A was negative; Ki67 was 5%. There were no signs of vascular or perineural invasion; the surgical margins were clear of disease. The three lymph nodes which were removed were free of tumor cells. Postoperatively, the patient developed a pancreatic fistula confirmed by an increase of drain output from the abdominal drainage and by high levels of amylase in the abdominal drain output, which was initially managed conservatively with dietary changes (low-fat diet) and octreotide therapy. Imaging showed a small fluid collection near the major pancreatic duct with no signs of peripancreatic abscesses. Endoscopic retrograde cholangiopancreatography (ERCP) was attempted 12 days later but failed to cannulate the pancreatic duct. A subsequent ERCP 9 days later showed a minor leakage from the main pancreatic duct into the adjacent fluid collection; conservative management was confirmed, and with dietary management and slow tapering of octreotide therapy, the fistula resolved in another 4 days, almost a month from the initial surgery. During follow-up, almost 2 months later, the patient developed acute pancreatitis, which was managed conservatively with no recurrence of fluid collections near the pancreas. However, an abdominal MRI showed a small hepatic lesion of 7 mm in segment VII–VI, hyperechoic with no other distinctive features, which was suspected to be a metastatic lesion and was subjected to strict radiological and clinical follow-up. The lesion grew during follow-up with a very slow growth rate, and 20 months after the initial surgery, it exceeded 1 cm on its longer axis. Furthermore, a similar lesion of 6 mm was now visible adjacent to the known lesion with similar radiological characteristics (Figure 2). The patient was admitted to the pediatric surgery ward, and after multidisciplinary discussion involving pediatric oncologists, adult pancreatic surgeons, and pediatric surgeons, she underwent a percutaneous ultrasound-guided liver biopsy that confirmed the metastatic nature of the lesion with immunohistochemical findings matching the primary lesion. A new laparotomy on the previous incision was performed; intraoperative ultrasound was used to localize the two lesions, which were confirmed to be between segment VII and VI. A Pringle maneuver was performed, and a 2.5 wedge of hepatic tissue encompassing both lesions was resected with adequate surgical margins. Biliary leakage on the resection margin was sutured, and hemostasis was achieved with haemostatic agents and fibrin sealant. One enlarged retroperitoneal lymph node was resected for histopathological analysis. Two abdominal drains were placed; the postoperative course was uneventful, with the patient resuming oral intake by the fourth postoperative day. Histological analysis of the resected lesions confirmed their metastatic nature with an immunohistochemical profile identical to the primary lesion and the tissue from the subsequent liver biopsy. Currently, the patient is undergoing regular follow-up (3 and a half year from the first surgery) with no evidence of recurrence.

### 3.2. Case 2

A 14-year-old female presented to the digestive surgery clinic with a visible and palpable abdominal mass in the epigastric and left hypochondriac region. The patient reported noticing this mass 3 months earlier and noticed that it had enlarged; she also lamented cramp-like abdominal pain in the last 3 weeks. For these reasons, she underwent abdominal ultrasound and abdominal MRI in an external center with evidence of a heterogeneous mass in the body and tail of the pancreas. Initial differential diagnosis based on imaging and clinical characteristics included mucinous cystic teratoma and SPN of the pancreas. The mass displaced the stomach and the spleen, mildly compressing the left kidney and causing slight aortic displacement with no radiological evidence of infiltration of adjacent structures. Based on these findings, the patient was admitted to the digestive surgery ward for surgical management. There was no evidence of distant metastasis or of lymph node involvement in a full body CT scan which the patient underwent after admission. She then underwent distal splenopancreatectomy. The surgical procedure was uneventful with complete tumor removal. Histological examination confirmed the diagnosis of SPN of the pancreas. Postoperatively, she exhibited normal glycemic profiles excluding the need for insulin therapy. By postoperative Day 8, increased drainage of milky fluids from the abdominal drain suggested a lymphatic fistula. Management was conservative with fasting associated with total parenteral nutrition and careful monitoring of the drain output which progressively decreased during 2 weeks. The patient resumed oral intake with a low-fat diet with no recurrence of lymphatic drainage, and successful removal of abdominal drains with no further intervention. Currently, the patient is undergoing regular follow-up (almost 2 and a half year from the first surgery) with no evidence of local or systemic recurrence.

## 4. Discussion

Even if some aspects regarding the management of SPNs, like criteria for malignancy and clinical meaning of actionable mutations, have not yet reached an international consensus, they are still regarded as generally benign lesions. The European evidence-based guidelines on pancreatic cystic neoplasms by The European Study Group on Cystic Tumours of the Pancreas from 2018 suggest an aggressive surgical approach even for locally advanced, metastatic, and recurrent cases (Grade 2C, strong agreement). Preoperative imaging is not yet capable of providing us a certain diagnosis, and a definitive diagnosis can only be acquired through anatomopathological examination after surgery; complete surgical resection is of paramount importance as not to miss focal signs of malignancy in the mass. When the anatomopathological diagnosis is achieved, there is no internationally shared recommendation for adjuvant (or neoadjuvant) chemotherapy [30].

Our cases have been managed with surgery alone, which was effective even in the presence of systemic recurrence with a good outcome, both in survival and in morbidity.

The recurrence of the tumor in the liver for one of our patients after 20 months from the initial surgery underlines the importance of accurate follow-up and the need for multidisciplinary management of these lesions in pediatric patients: Pediatric oncologists and pediatric surgeons should discuss the nature of these lesions together with the anatomopathologist and with surgeons that usually treat adult patients with pancreatic neoplasms as to define the better surgical treatment and follow-up plan.

The following features are more common for pediatric SPNs: (1) They predominantly affect young adolescent females, (2) the primary lesion can be very large at diagnosis, (3) despite the large masses at presentation, few patients had distant metastases or anatomopathological signs of infiltration of adjacent structures or of lympho-vascular invasion, (4) distant metastases are rare, and most lesions are resectable, and (5) patients who undergo complete resection have a high long-term survival rate even in the case of secondary surgery for metachronous metastasis.

While SPNs of the pancreas are lesions with the potential to metastasize, its removal offers an excellent chance of survival compared to other pancreatic neoplasms like adenocarcinoma and pancreatic neuroendocrine tumors. Surgery is still considered the gold standard for treating localized SPNs and is linked to increased overall survival..

The type of surgery needed for treating these lesions depends on its location, size, and how it relates to adjacent structures. While most tumors are usually treated with open surgery, a recent systematic review has shown that minimally invasive pancreatectomy offers several advantages over the traditional open approach [7, 31, 32]. These advantages include less intraoperative blood loss, reduced need for blood transfusions, shorter postoperative dietary restrictions, and an overall shorter hospital stay. Laparoscopic pancreatic surgery, in particular, is a relatively new approach that is currently being studied as a possible alternative to open surgery. While laparoscopic distal pancreatectomy is widely performed in many centers, more complex types of resection, such as Whipple's procedure or central pancreatectomy, are only carried out laparoscopically in highly specialized centers [1, 33–35].

Depending on the tumor's location, various types of surgeries can be performed, including distal pancreatectomy with or without splenectomy, pancreatoduodenectomy with pylorus preservation, and Whipple's procedure. Enucleation may be an option for small SPNs located far from the pancreatic duct, but it is linked to a high risk of developing a pancreatic fistula [36]. About 10% of SPT cases lead to metastasis in the liver or peritoneum. In such situations, metastasectomy is recommended either at the time of primary resection or for recurrences whenever complete surgical removal is possible [9, 37–41].

## 5. Conclusions

Both cases we have described underwent surgical resection, both as primary treatment at the moment of the diagnosis of a pancreatic mass and as rescue therapy for liver metastasis. The first case involved the enucleation of the lesion at the *cefalo*-pancreatic site and the secondary atypical liver resection of the sixth to seventh segment; as previously described in literature, enucleation determined the formation of a pancreatic fistula in the postoperative period. Even if management was conservative, the risk of enucleations should always be weighed against the removal of more healthy pancreatic mass and its metabolic implications (e.g., possible worse glucose tolerance and development of diabetes after removal of large volume of pancreas mass). The second case underwent distal splenopancreasectomy and did not require further intervention with both the surgery and the postoperative period being uneventful. In both cases, a laparotomic approach was performed. The experience of our center has confirmed that wide margin surgery, with the option of metastasectomy if necessary, is the optimal treatment for this type of neoplasm guaranteeing effective control of the disease even at recurrence. Even if the primary lesion is very large, surgery should always be the primary treatment option. Based on our experience, ultrasound follow-up has proven to be appropriate for early detection of recurrences and metastases and should always be used when other radiological exams are not definitive and whenever there is a need for more strict follow-up with the intent to spare radiations in these patients who are frequently female and fertile.
